# Genome-wide association study and polygenic risk scores of retinal thickness across the cognitive continuum: data from the NORFACE cohort

**DOI:** 10.1186/s13195-024-01398-8

**Published:** 2024-02-16

**Authors:** María Eugenia Sáez, Ainhoa García-Sánchez, Itziar de Rojas, Emilio Alarcón-Martín, Joan Martínez, Amanda Cano, Pablo García-González, Raquel Puerta, Clàudia Olivé, Maria Capdevila, Fernando García-Gutiérrez, Miguel Castilla-Martí, Luis Castilla-Martí, Ana Espinosa, Montserrat Alegret, Mario Ricciardi, Vanesa Pytel, Sergi Valero, Lluís Tárraga, Mercè Boada, Agustín Ruiz, Marta Marquié

**Affiliations:** 1 Ace Alzheimer Center Barcelona, Universitat Internacional de Catalunya (UIC), Barcelona, Spain; 2 Centro Andaluz de Estudios Bioinformáticos (CAEBI), Seville, Spain; 3 Networking Research Center on Neurodegenerative Diseases (CIBERNED), Instituto de Salud Carlos III, Madrid, Spain; 4 Clínica Oftalmológica Dr. Castilla, Barcelona, Spain; 5 Vista Alpina Eye Clinic, Visp, Switzerland; 6 PhD Programme in Surgery and Morphological Sciences, Universitat Autònoma de Barcelona, Barcelona, Spain; 7 Hôpital ophtalmique Jules-Gonin, Fondation asiles des aveugles, University of Lausanne, Lausanne, Switzerland; 8 Biggs Institute for Alzheimer’s and Neurodegenerative Diseases, University of Texas Health Science Center, San Antonio, Texas USA

**Keywords:** Alzheimer’s disease (AD), Optical coherence tomography (OCT), Genome-wide association study (GWAS), Polygenic risk score (PRS), Mendelian randomization (MR), NORFACE, GR@CE

## Abstract

**Background:**

Several studies have reported a relationship between retinal thickness and dementia. Therefore, optical coherence tomography (OCT) has been proposed as an early diagnosis method for Alzheimer’s disease (AD). In this study, we performed a genome-wide association study (GWAS) aimed at identifying genes associated with retinal nerve fiber layer (RNFL) and ganglion cell inner plexiform layer (GCIPL) thickness assessed by OCT and exploring the relationships between the spectrum of cognitive decline (including AD and non-AD cases) and retinal thickness.

**Methods:**

RNFL and GCIPL thickness at the macula were determined using two different OCT devices (Triton and Maestro). These determinations were tested for association with common single nucleotide polymorphism (SNPs) using adjusted linear regression models and combined using meta-analysis methods. Polygenic risk scores (PRSs) for retinal thickness and AD were generated.

**Results:**

Several genetic *loci* affecting retinal thickness were identified across the genome in accordance with previous reports. The genetic overlap between retinal thickness and dementia, however, was weak and limited to the GCIPL layer; only those observable with all-type dementia cases were considered.

**Conclusions:**

Our study does not support the existence of a genetic link between dementia and retinal thickness.

**Supplementary Information:**

The online version contains supplementary material available at 10.1186/s13195-024-01398-8.

## Background

Alzheimer’s disease (AD) is the most common form of dementia in the elderly, responsible for 60–80% of cases. AD is a slowly progressive neurodegenerative condition that irreversibly impairs cognition and results in a complete loss of autonomy [1]. Its main neuropathological hallmarks are β-amyloid plaques and neurofibrillary tangles composed of hyper-phosphorylated tau [2]. It is known that these brain changes start developing up to two decades before the dementia onset, and, therefore, the identification of early biological markers of the disease is currently the focus of intensive research.

Nowadays, it is possible to detect these AD-core neuropathological changes while individuals are alive and have not yet developed dementia by using different imaging and fluid biomarker techniques, such as positron emission tomography, magnetic resonance imaging, and cerebrospinal fluid [3]. The currently available biomarkers are either expensive or invasive, and, thus, there is great interest in identifying novel ones that are sensitive and specific to early AD changes but also easy to administer, inexpensive, non-invasive, and widely accessible. Research efforts are now focused on the fields of plasma, genomics, and retinal imaging, among others [4–6].

The retina is the neuronal structure of the eye and is embryologically derived from the diencephalon, as is the optic nerve, and both are considered part of the central nervous system [7]. Retinal structures can be visualized in vivo using non-invasive high-resolution imaging methods, such as optical coherence tomography (OCT), which has been employed for decades in the ophthalmology field to diagnose and monitor common ocular pathologies, such as glaucoma, diabetes retinopathy, and age-related macular degeneration [8]. In the past few years, changes in thickness and volume of different retinal layers have also been observed in several neurological disorders, such as optic neuritis, multiple sclerosis, Parkinson’s disease, and AD [9]. In AD in particular, degeneration of the retinal nerve fiber layer (RNFL) and ganglion cell layer (GCL) has been observed in postmortem tissue [10]. Multiple publications have shown that patients with AD dementia (ADD) and mild cognitive impairment (MCI) present RNFL and GCL thinning compared to healthy controls, as measured in vivo by OCT, although other articles have shown no significant differences [11, 12]. Interestingly, a recent study reported that both retinal structural and vascular measures were significantly decreased in dementia patients vs. cognitively healthy (CH) and MCI individuals but found no differences between amyloid positive and negative dementia patients [13].

Recent advances in the field of AD genetics [6, 14] have permitted the identification of several genetic factors associated with AD risk. To date, up to 82 different AD loci have been uncovered. Furthermore, the linear combination of existing loci using polygenic risk scores (PRS) has been demonstrated to be instrumental in the identification of high-risk populations and as a promising tool for selecting patients for clinical trials [6].

Regarding the association of genomics with retinal structures, several recent genome-wide association studies (GWAS) have identified genetic *loci* determining retinal thickness [15–17]. First, a GWAS using OCT images from 68,423 participants from the UK Biobank cohort identified 139 genetic loci associated with macular thickness, the most significant ones being highly expressed in the retina [16]. In another GWAS also using OCT images from 31,434 UK Biobank participants, 46 genetic loci associated with the thickness of the RNFL or ganglion cell inner plexiform layer (GCIPL, GCL + inner plexiform layer) were established, three of which were related to foveal hypoplasia and visual acuity [15]. More recently, Currant et al. have also described 111 loci associated with the thickness of one or more of the photoreceptor cell layers in a GWAS using OCT images from 31,135 participants from the UK Biobank [17], with a significant enrichment of genes involved in rare eye pathologies, such as retinitis pigmentosa.

In the present study, we performed a GWAS aimed at identifying genes associated with RNFL and GCIPL thickness assessed by OCT in a Spanish population with different degrees of cognitive impairment, including CH, MCI, and dementia (most of them ADD), evaluated in a memory clinic. We also constructed polygenic risk scores (PRS) for retinal thickness and AD and explored their association with RNFL and GCIPL thickness, as well as with cognitive status.

## Methods

### Study population

The study population comprised 3170 participants from the Neuro-Ophthalmology Research at Fundació ACE (NORFACE) cohort, which was founded in 2014 to search for retinal biomarkers of AD and examine the relationships between retinal changes and different types of neurodegenerative disorders [12]. Consecutive individuals evaluated due to cognitive decline at Ace Alzheimer Center Barcelona between September 2014 and March 2019 with a diagnosis of CH, MCI, or dementia, and who had available GWAS data from the GR@ACE study [18] were included in the present analysis.

Participants were recruited from four sources within Ace Alzheimer Center Barcelona (ACE): (1) the Memory Clinic, an outpatient diagnostic unit for individuals with cognitive decline that has an agreement with the Catalan Public Health System [19], (2) Ace’s Open House Initiative [20], a social program that provides free assessment of the cognitive status of individuals from the community without the need for a physician’s referral, (3) the Fundació ACE Healthy Brain Initiative (FACEHBI), a longitudinal research study of aging, lifestyle, cognition, and biomarkers in individuals with subjective cognitive decline (SCD) [21], and (4) the BIOFACE project, a research study of novel biomarkers focused on individuals with early-onset MCI [22].

### Clinical diagnostic groups

Study participants completed neurological, neuropsychological, and social evaluations at ACE. For each individual, a consensus-based diagnosis of cognitive status was reached at the time of study recruitment by a multidisciplinary team of professionals that included neurologists, neuropsychologists, and social workers [19].

Dementia was defined according to the DSM-V criteria [23]. Within the dementia group, ADD [1] represented 84.3% of cases, followed by vascular dementia [24] (8.8%), while the remaining 6.9% included other types of dementia, such as dementia with Lewy bodies [25] (2.2%) and frontotemporal [26] (0.9%). MCI was defined using Petersen’s [27] and the Cardiovascular Health and Cognition Study criteria [28]. All individuals in the CH group had a Clinical Dementia Rating (CDR) score [29] of 0, a preserved performance (score ≥ 27) on the Mini-Mental State Examination (MMSE) [30, 31] and a strictly normal performance on the neuropsychological battery of Fundació ACE (NBACE) [32, 33].

### Neuro-ophthalmological evaluation

In parallel with the cognitive assessment, study participants underwent a complete neuro-ophthalmological evaluation, which lasted about 20 min and was performed by an optometrist. The evaluation comprised (1) a review of past ophthalmological diseases, treatments, and surgeries, (2) a monocular visual acuity assessment, with the participants wearing their habitual correction for refractive error using a pinhole occluder and the Early Treatment of Diabetic Retinopathy Study (ETDRS) chart [34, 35], and (3) an intraocular pressure (IOP) measurement by Icare tonometry [36]. More details can be found elsewhere [37]. The ophthalmologist and neurologists were blind to each other’s diagnoses.

### Optical coherence tomography

Participants were imaged with a 3D-OCT Maestro® (Fast Map software version 8.40) and/or a DRI OCT Triton—Swept Source (SS) OCT (software v.1.22.1; Topcon Co. Tokyo, Japan). In the final cohort, 1789 individuals were scanned with the OCT Maestro, 1123 with the OCT Triton, and 582 with both OCT devices performed the same day. The OCT exam was completed in about 5–10 min. Both eyes were scanned separately. No pupil dilation was required. Retinal layer segmentation was performed using the Topcon Advanced Boundary Segmentation TM (TABS) algorithm as part of the Fast Map software [38]. Data from RNFL and GCIPL thickness at the macula were analyzed.

### Statistical analysis

RNFL and GCIPL thickness measures were scaled before analysis. Correlation between macular RNFL and GCIPL thickness by eye and OCT dataset were explored by means of Spearman correlation analysis. Correlogram plots were generated using the corrplot R package [39].

The associations of RNFL and GCIPL thickness measures with cognitive status were explored by means of logistic regression models adjusted by the OCT image quality parameter provided by the OCT device, age, sex, years of education, APOE genotype, genotyping batch (I/II), and concomitant ocular diseases/surgeries. A meta-analysis of the Maestro and Triton OCT cohorts was run using meta R package [40] and a fixed effect model. *P* values below the 0.05 threshold were considered significant.

### Genome-wide association study (GWAS)

Genotyping was conducted using the Axiom 815 K Spanish biobank array (Thermo Fisher) at the Spanish National Center for Genotyping (CeGEN, Santiago de Compostela, Spain), which contains rare population-specific variations observed in the Spanish population. Genotyping, quality control (QC), and imputation procedures have been described elsewhere [18]. For the OCT Triton dataset, 1465 samples were available after QC; for the OCT Maestro cohort, after QC and exclusion of samples included in the Triton dataset, 1705 remained available for GWAS.

Association analyses between single-nucleotide polymorphisms (SNPs) and retinal thickness measurements were performed independently by each eye (left, right) and OCT device (Maestro, Triton) using a linear regression procedure, assuming an additive model in Plink 2 [41]. Only SNPs with minor allele frequency (MAF) above 0.01 were kept for analysis. All analyses were adjusted by age, sex, genotyping batch (I/II), and concomitant ocular diseases/surgeries that could affect retinal thickness, OCT image quality, the first 10 principal component (PC) vectors, and cognitive status (dementia, MCI, or CH). Ocular conditions included as adjusting factors in the model were the following: open angle glaucoma, maculopathy, retinal surgery, amblyopia, high myopia (< −6Dp) or hyperopia (> +6Dp), and intraocular pressure > 24 mmHg. The top results were aggregated by genomic region and linkage disequilibrium blocks using the clump procedure implemented in Plink 2. Additionally, an interaction term with dementia status (presence vs. absence) was included to assess whether the presence of the disease modulated the association of the SNPs with the retinal thickness measurements. The fixed-effects meta-analysis procedures implemented in Plink 2 were used to combine the association analysis from both datasets and both eyes.

The GWAS significance threshold was established in *p* = 5e−8; *p* values below *p* = 10e−5 were considered suggestive of association. The genomic inflation factor (*λ*) was calculated as the median of the resulting chi-square test statistics divided by the expected median of the chi-square distribution. Manhattan and QQ plots were generated using qqman R package [42].

### Functional analyses

An over-representation analysis was performed, aimed at identifying the Gene Ontology (GO) categories and signaling pathways using gprofiler2 R package [43].

### Mendelian randomization (MR) analysis

With the aim or investigating the causal relationships between retinal structures and dementia, we calculated individual polygenic risk scores (PRS) for RNFL and GCIPL thickness in the full GR@ACE cohort (*N* = 17,089, including 5195 ADD patients, 2509 non-AD dementia patients, 794 MCI, and 8579 CH) based on the genetic variants below the genome-wide significance threshold described by [15]. For RNFL, 23 available SNPs of the 30 reported by Currant et al. were used to compute the PRS, while, for GCIPL, the full 22-SNP set was used. Similarly, the PRS for AD was calculated in the study sample using 83 SNPs showing genome-wide significant association with AD according to [14].

Additive PRSs were computed for AD, RNFL, and GCIPL as ${\sum }_{i=0}^{n}\beta x SNPij$, where *β* is the weight for each of the variants in the GWAS, and $SNPij$ is the number of alleles (0–2 range). PRSs were scaled prior to analysis.

Spearman correlation and linear regression were used to explore the relationship between AD PRS and retinal PRSs (RNFL PRS and GCIPL PRS) and for retinal determinations and AD PRs and retinal PRSs. The associations of AD PRS, RNFL PRS, and GCIPL PRS with cognitive status were explored by means of logistic regression analysis. All regression models were adjusted for age, sex, APOE genotype, years of education, genotyping batch (I/II), and concomitant ocular diseases; regression models for RNFL or GCIPL thickness and AD status were additionally adjusted by the quality of retinal measures. Meta-analysis of the OCT Maestro and Triton cohorts was performed using meta R package. A *p* value < 0.05 was considered significant in this analysis.

## Results

### Study population and retinal measurements

The study population included 1847 dementia patients, 764 MCI patients, and 559 CH individuals from the NORFACE cohort (Table 1). As expected, dementia cases were older and had lower MMSE scores and fewer years of education but a higher prevalence of the APOE E4 allele and a larger proportion of females.

**Table 1:** Characteristics of the study population

	**Dementia (*N* = 1847)**	**MCI (*N* = 764)**	**HC (*N* = 559)**	**Total (*N* = 3170)**	***P* value**	**ADD (*N* = 1554)**	**Non-ADD (*N* = 293)**	***P* value**
**Sex**					< 0.001			< 0.001
Male	547 (29.6%)	320 (41.9%)	180 (32.2%)	1047 (33.0%)		430 (27.7%)	117 (39.9%)	
Female	1300 (70.4%)	444 (58.1%)	379 (67.8%)	2123 (67.0%)		1124 (72.3%)	176 (60.1%)	
**Age (years)**					< 0.001			0.305
*N*	1847	764	559	3170		1554	293	
Median [Q1,Q3]	82.0 [77.0, 86.0]	74.0 [69.0, 80.0]	65.0 [59.0, 70.0]	78.0 [70.0, 84.0]		82.0 [77.0,86.0]	81.0 [76.0,86.0]	
Mean (sd)	80.72 (7.31)	64.57 (8.64)	73.55 (8.55)	76.14 (9.97)		80.8 (7.3)	80.3 (7.4)	
**Education (years)**					< 0.001			0.459
*N*	1784	721	295	2800		1501	283	
Median [Q1,Q3]	6.0 [3.0, 8.0]	6.0 [5.0, 10.0]	10.0 [8.0, 14.5]	6.0 [3.0, 10.0]		6.00 [3.0,8.0]	6.00 [3.0,8.0]	
Mean (sd)	5.93 (4.18)	11.39 (4.23)	7.55 (4.37)	6.92 (4.56)		5.9 (4.1)	6.2 (4.5)	
**MMSE score**					< 0.001			< 0.001
*N*	1837	761	557	3155		1544	293	
Median [Q1,Q3]	20.0 [17.0,23.0]	26.0 [24.0, 28.0]	30.0 [29.0, 30.0]	23.0 [19.0, 28.0]		19.0 [16.0, 23.0]	22.0 [18.0, 25.0]	
Mean (sd)	19.45 (4.75)	29.41 (0.86)	25.67 (3.11)	22.71 (5.65)		19.1 (4.8)	21.3 (4.3)	
**APOE genotype**					< 0.001			< 0.001
NA	7 (0.4%)	8 (1.0%)	1 (0.2%)	16 (0.5%)		5 (0.3%)	2 (0.7%)	
e2e2	7 (0.4%)	6 (0.8%)	1 (0.2%)	14 (0.4%)		5 (0.3%)	2 (0.7%)	
e2e3	105 (5.7%)	51 (6.7%)	63 (11.3%)	219 (6.9%)		81 (5.2%)	24 (8.2%)	
e2e4	29 (1.6%)	12 (1.6%)	11 (2.0%)	52 (1.6%)		27 (1.7%)	2 (0.7%)	
e3e3	985 (53.3%)	446 (58.4%)	348 (62.3%)	1779 (56.1%)		803 (51.7%)	182 (62.1%)	
e3e4	621 (33.6%)	211 (27.6%)	122 (21.8%)	954 (30.1%)		548 (35.3%)	73 (24.9%)	
e4e4	93 (5.0%)	30 (3.9%)	13 (2.3%)	136 (4.3%)		85 (5.5%)	8 (2.7%)	
**Comorbidities**^**a**^								
Amblyopia	33(1.8%)/31(1.7%)	14(1.8%)/24(3.1%)	17(3.0%)/10(1.8%)	64(2.0%)/65(2.1%)	0.166/0.050	29(1.9%)/27(1.7%)	4(1.4%)/4(1.4%)	0.553/0.649
Surgery	33(1.8%)/35(1.9%)	34(4.5%)/39(5.1%)	34(6.1%)/35(6.3%)	101(3.2%)/109(3.4%)	< 0.001/ < 0.001	24(1.5%)/27(1.7%)	9(3.1%)/8(2.7%)	0.070/0.253
AMD	22(1.2%)/24(1.3%)	12(1.6%)/12(1.6%)	0(0.0%)/2(0.4%)	34(1.1%)/38(1.2%)	0.017/0.111	19 (1.2%)/21(1.4%)	3(1.0%)/3(1.0%)	0.774/0.650
Dps < -6 or > + 6	2(0.1%)/2(0.1%)	0(0.0%)/0(0.0%)	0(0.0%)/0(0.0%)	2(0.1%)/2(0.1%)	0.488/0.488	2(0.1%)/2 (0.1%)	0(0.0%)/0(0.0%)	0.539/0.539
Glaucoma	74(4.0%)/77(4.2%)	38(5.0%)/38(5.0%)	23(4.1%)/22(3.9%)	135(4.3%)/137(4.3%)	0.529/0.579	60(3.9%)/63(4.1%)	14(4.8%)/14(4.8%)	0.463/0.569
IOP > 24 mmHg	30(1.6%)/29(1.6%)	13(1.7%)/19(2.5%)	14(2.5%)/14(2.5%)	57(1.8%)/62(2.0%)	0.380/0.180	24(1.5%)/21(1.4%)	6(2.0%)/8(2.7%)	0.532/0.082
Other	8(0.4%)/8(0.4%)	7(0.9%)/5(0.7%)	3(0.5%)/3(0.5%)	18(0.6%)/16(0.5%)	0.325/0.763	8(0.5%)/7(0.5%)	0(0.0%)/1(0.3%)	0.218/0.794
**GCIPL**^**a**^					< 0.001/ < 0.001			0.127/0.842
*N*	1727/1765	700/713	529/536	2956/3004		1446/1470	281/285	
Median [Q1,Q3]	61.5[55.8,66.5]/61.6[55.3,66.4]	64.4[59.0,69.4]/64.6[58.8,69.5]	64.4[60.6,68.1]/64.0[60.5,68.1]	62.8[57.4,67.5]/62.7[57.0,67.5]		61.3[55.7,66.4]/61.68[55.2,66.4]	62.2[56.2,67.0]/61.2[55.9,66.5]	
Mean (sd)	59.45(14.03)/58.47(14.85)	62.85(10.29)/62.67(10.27)	62.37(11.94)/62.2(12.72)	60.75(13.04)/60.11(13.77)		59.3(14.3)/58.4(14.8)	60.5(12.6)/58.7(15.3)	
**RNFL**^**a**^					< 0.001/ < 0.001			0.111/0.021
*N*	1727/1755	700/713	529/536	2956/3004		1446/1470	281/285	
Median [Q1,Q3]	35.6[31.9,40.3]/36.5[32.6,42.0]	36.0[33.0,40.1]/36.7[33.3,41.6]	37.4[34.5,41.1]/38.7[35.1,42.6]	36.1[32.7,40.4]/37.0[33.3,42.0]		35.70[31.9,40.4]/36.6[32.9,42.20]	35.10[31.50,39.5]/35.8[31.7,41.0]	
Mean (sd)	39.36(20.78)/40.09(21.14)	41.23(17.26)/42.13(17.95)	41.46(20.39)/41.96(20.8)	40.19(20.12)/40.9(20.54)		39.8(21.7)/40.7(21.8)	37.0(15.4)/37.2(17.1)	

*AMD* Age-related macular degeneration, *Dps* Dioptres, *IOP* Intraocular pressure, *MMSE* Mini-Mental State Examination, *APOE* Apolipoprotein E, *GCIPL* Ganglion cell inner plexiform layer, *RNFL* Retinal neve fiber layer, *sd* Standard deviation

^a^Right /left eye

**Fig. 1:**
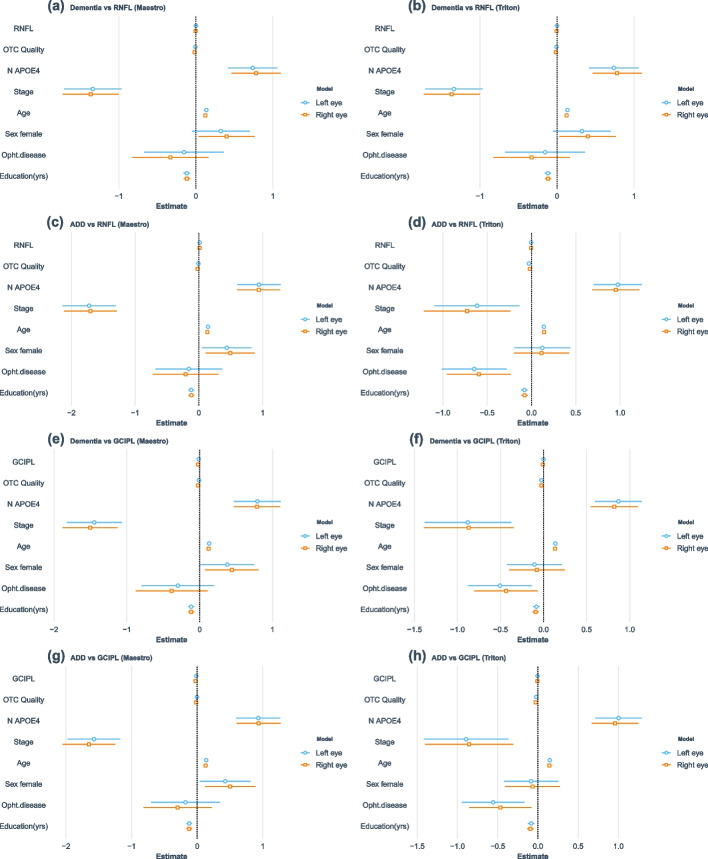
Regression models for all-type dementia and ADD vs. retinal thickness. Beta regression and standard error (SE) are represented

The analysis of raw RNFL and GCIPL thickness showed significant differences among diagnostic groups, with dementia patients showing the lowest median values for both retinal measures in both eyes. In the adjusted logistic models including only ADD cases, however, retinal measurements were not significantly associated with dementia status, with the exception of GCIPL measurements in the OCT Maestro cohort, although with a reduced effect size (Fig. 1). After meta-analysis, the association of GCIPL thickness with dementia remained significant, with a similar effect in both eyes (*β* = 0.98, *p* = 1e−4 and *β* = 0.98, *p* = 1.2e−5 for left and right eye, respectively); exclusion of non-AD dementia patients generated similar results (*β* = 0.98, *p* = 5.6e−6 and *β* = 0.98, *p* = 2.1e−6 for left/right eye).

Correlation analysis in individuals with retinal measures from both OCT devices (*N* = 582) showed a strong correlation between left and right RNFL thickness measures (*r* = 0.89 for OCT Maestro, *r* = 0.63 OCT for Triton; Supplementary Fig. 1). Similarly, left and right GCIPL measures were also strongly correlated in both OCT datasets (*r* = 0.77 for OCT Maestro and *r* = 0.82 for OCT Triton). By contrast, correlations of retinal thickness measures between the two OCT devices were much lower, in the 0.26–0.40 range. Interestingly, while in the OCT Triton a positive correlation between GCIPL and RNFL thickness measures was observed, these two determinations were inversely correlated in the OCT Maestro cohort.

### Genome-wide association study (GWAS)

A GWAS of RNFL and GCIPL thickness was performed independently by OCT cohort and eye, then results from both OCT devices were combined by eye; suggestive signals from the left eye (*p* < e−5) were explored for replication in the right eye, with the aim of filtering out spurious associations.

**Fig. 2:**
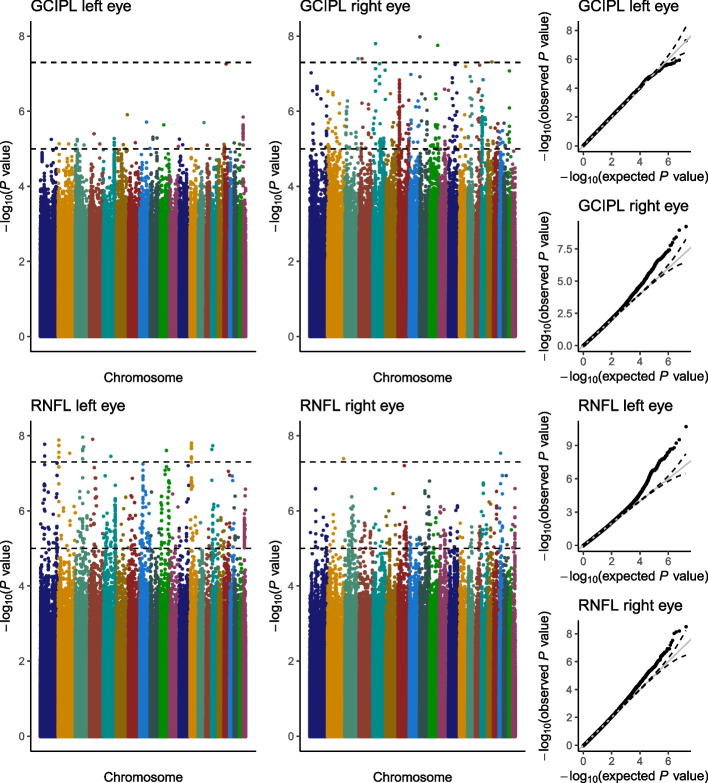
Meta-analysis of GCIPL and RNFL thickness GWAS (both eyes). Manhattan plots representing log_10_*p* values from adjusted PLINK linear regression models by genomic location

For GCIPL thickness, the meta-analysis of left eye OCT Triton and OCT Maestro GWAS results (*λ* = 1.01) identified 72 suggestive signals with a single SNP on chromosome 18, rs147136024, showing borderline genome-wide significance (*p* = 5.78 × 10 − 8; Fig. 2). When compared with results from the right eye (*λ* = 0.99), 69 SNPs were consistently associated in both eyes; these SNPs were clustered in 26 suggestive independent *loci* (top SNP *p* < 5e−8) showing similar effects across OCT datasets and eyes, reaching 7 of genome-wide significance in the combined meta-analysis (Table 2, Supplementary Table 1). Of note, two intragenic regions showed the largest evidence of association: a 13.6-kb region on chromosome 5q33.1, encoding two lncRNA genes with opposite transcription directions (LINC01470 and ENSG00000286749), and a 57.7-kb region on chromosome 22q11.1 around TPTE pseudogene 1 (TPTEP1). Other suggestive signals were located near *loci* previously associated with retinal features, myopia, or abnormality of refraction, such as the TSPAN10/NPLOC4/PDE6G locus or the retinoid acid receptor beta RARB, associated with retinal vasculature in a previous GWAS. Enrichment analysis showed an over-representation of molecules with binding properties, many of them involved in transport across membranes.

**Table 2:** GCL and RNFL thickness meta-analysis: top concordant signals across datasets and eyes

**Top SNP**	**RS**	**Total SNPs**	**kb**	**A1**	***P* meta**	**BETA**	***Q***	***I***	**Triton**		**Maestro**		**Nearest genes (200 kb)**
									**Left eye**	**Right eye**	**Left eye**	**Right eye**	
**GCIPL thickness**													
5:152625167:A:C	rs11167584	17	13.6	C	2.3e−10	−1.31	7.6e−01	0	−1.24	−1.68	−1.25	−1.04	LOC101927134(0)
3:120804007:C:CG	rs373444630	1	0.0	CG	5.3e−09	1.07	9.9e−01	0	1.06	1.09	1.17	0.96	GTF2E1(+20.9 kb)| STXBP5L(−104.2 kb)
17:81648542:A:G	rs7405453	1	0.0	A	5.9e−09	−1.06	3.7e−02	64.8	−1.5	−1.49	−0.41	−0.42	TSPAN10(0)
6:154594197:T:C	rs2139855	1	0.0	T	9.5e−09	−1.03	7.5e−01	0	−1.3	−0.81	−0.96	−0.92	CNKSR3(+83.6 kb)|SCAF8(−139.2 kb)
2:14362480:A:G	rs182487621	1	0.0	G	1.3e−08	−4.53	9.8e−01	0	−4.65	−3.92	−4.94	−4.66	LINC00276(0)
22:16612526:C:T	rs11089268	25	57.7	T	3.7e−08	−1.19	8.2e−01	0	−1.22	−0.92	−1.52	−1.1	TPTEP1(0)
18:27756533:T:C	rs147136024	1	0.0	C	4.1e−08	−4.2	4.2e−02	63.5	−7.02	−3.49	−3.13	−1.27	CDH2(−194.4 kb)
**RNFL thickness**													
1:110872670:C:T	rs61803955	4	2.7	T	1.0e−10	5.6	5.6e−02	60.34	6.3	3.6	8.2	12.0	CD53(0)
1:60043219:G:A	rs149444168	7	231.1	A	2.1e−10	5.6	9.0e−03	74.08	7.9	2.5	7.6	10.7	C1orf87(0)
5:105525900:A:G	NA	3	103.3	G	9.2e−10	5.8	2.4e−01	28.3	8.2	4.2	4.7	4.6	
8:42885667:A:C	rs143666229	3	139.4	C	4.9e−09	4.9	1.2e−01	49.03	7.4	3.4	2.6	2.6	RNF170(0)
18:77673191:C:T	rs512467	4	1.3	C	5.2e−09	1.3	1.2e−01	47.93	2.0	0.9	0.6	1.2	
1:121516475:G:C	rs114500301	3	114.8	C	6.2e−09	5.7	5.7e−01	0	7.4	5.0	3.9	3.6	EMBP1(−2.6 kb)|SRGAP2-AS1(+118.6 kb)
12:24137871:T:C	rs12372308	2	0.0	C	7.3e−09	3.6	3.8e−03	77.63	5.4	1.2	5.3	7.6	SOX5(0)
8:43806675:G:A	rs12681971	9	145.9	A	1.1e−08	4.8	1.5e−01	43.6	7.1	3.4	2.7	2.3	
2:9674766:C:G	rs16867115	10	9.4	G	1.5e−08	4.9	6.6e−02	58.25	7.8	2.7	3.8	6.1	TAF1B(−168.7 kb)|YWHAQ(+43.71 kb)
10:132126425:C:T	rs2814187	2	0.0	C	1.5e−08	−1.2	1.7e−01	39.82	−1.5	−0.7	−2.2	−1.4	JAKMIP3(0)
20:14267811:T:C	rs6105236	9	11.8	C	1.6e−08	1.5	8.8e−02	54.1	1.9	0.8	2.3	2.8	MACROD2(0)
3:107833982:A:G	rs116535715	2	0.0	G	1.6e−08	5.2	3.9e−03	77.54	7.2	2.0	13.4	9.0	LINC00635(−7.7 kb)|LINC00636(−49.2 kb)
11:43040082:C:T	rs148342611	3	57.8	T	3.6e−08	5.7	1.9e−01	36.21	8.1	3.4	5.3	7.2	
13:38256057:A:C	rs564689	49	28.1	C	4.0e−08	5.3	5.1e−02	61.48	8.7	2.9	5.0	3.5	LINC00571(+112.8 kb)|UFM1(−93.7 kb)

*Kb* Kilobases, *Q P*-value for Cochrane’s Q statistic, *I I*^2^ heterogeneity index (0–100)

Left eye meta-analysis of OCT Triton and Maestro RNFL thickness genome-wide association results (*λ* = 0.98) identified 609 SNPs suggestive of association, 45 below the genome-wide significance threshold (Fig. 2). Only 388 of these SNPs showed the same direction of effect in all the analyses, representing 71 suggestive genome regions, including PTPRD, previously associated with RNFL, or FGFR2 and FHIT, both associated with retinal vasculature (Table 2, Supplementary Table 1). Of these regions, 14 reached the 5e−8 threshold after meta-analysis, including a 28.1-kb region on 13q13.3 overlapping the LINC00571 gene, a lncRNA gene previously associated with myopia, schizophrenia, and, interestingly, educational attainment and tau measures. Signals arising from the APOC1/APOE/TOMM40 locus were also observed on the left eye meta-analysis but showed opposite effect size directions in the OCT Triton and Maestro cohorts on the right eye, not reaching even the suggestive significance threshold on final RNFL thickness meta-analysis of both eyes. Enrichment analysis also highlighted proteins with binding properties involved in cellular metabolism.

We did not find overlap between RNFL and GCIPL thickness variants. We found no evidence of interaction between variants associated with either RNFL or GCIPL and either all-type dementia or ADD.

### Polygenic risk scores and Mendelian randomization

#### Correlation between retinal PRSs and retinal thickness measures

**Fig. 3:**
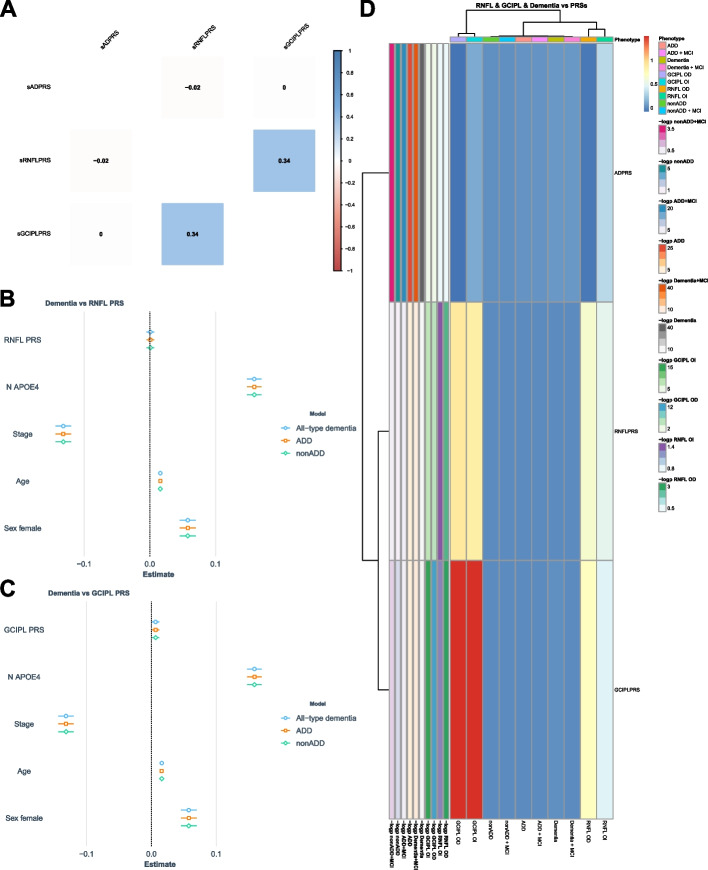
Polygenic risk scores (AD PRS, RNFL PRS, and GCIPL PRS). **A** Pearson’s correlation coefficient between PRSs (GR@ACE). **B** Adjusted regression models for RNFL PRS as predictor of dementia (GR@ACE). **C** Adjusted regression models for GCIPL PRS as predictor of dementia (GR@ACE). **D** Adjusted regression models for RNFL PRS, GCIPL PRS, and AD PRS as predictors of retinal thickness (meta-analysis Maestro and Triton) and dementia (GR@ACE); beta regression coefficients and log_10_*p* values are shown. OI: left eye; OD: right eye

RNFL and GCIPL PRSs showed significant but weak correlations with RNFL and GCIPL thickness measures in the range of *ρ* = 0.06–0.09 for RNFL PRS vs RNFL thickness to *ρ* = 0.16–0.23 for GCIPL PRS and GCIPL thickness (Supplementary Fig. 2). Adjusted models confirmed the association of retinal PRSs with retinal thickness measures, while AD PRS was not associated with RNFL or GCIPL thickness (Fig. 3D, Supplementary Fig. 3, Supplementary Table 3). We then investigated whether any of the SNPs reported by Bellenguez et al. included in the AD PRS were associated with RNFL or GCIPL measures at the nominal significance level of 0.05 by fitting adjusted linear regression models with allelic dosage as a predictor variable (Supplementary Tables 4–7, Supplementary Figs. 4–6). After a meta-analysis of the Maestro and Triton OCT results, only the rs587709 SNP, located within the leukocyte immunoglobulin-like receptor (LIR) cluster on 19q13.4, was consistently associated with RNFL thickness in both eyes, while two SNPs, rs7384878 (SPDYE3) and rs6586028 (TSPAN14), were associated with GCIPL thickness in both eyes.

#### Correlation between retinal PRSs and AD PRS

Aiming to further explore the relationship between cognitive status and retinal thickness, AD, RNFL, and GCIPL PRSs were calculated in the entire GR@ACE cohort, which comprises more than 17,000 individuals. Correlation between RNFL PRS and GCIPL PRS was 0.34 (Fig. 3A) in the GR@ACE dataset, with little variation according to cognitive status (Supplementary Fig. 7). The AD and RNFL PRSs showed a weak negative correlation in the entire cohort (*ρ* = −0.019, *p* = 0.014); in the stratified analysis by cognitive status, the largest absolute correlation was observed among MCI patients (*ρ* = −0.072, *p* = 0.0473), followed by non-AD dementia cases (*ρ* = −0.049) and CH (*ρ* = −0.026), while this correlation was positive in the ADD group (*ρ* = +0.021), although it did not reach statistical significance (*p* = 0.13). No significant correlations were observed between AD PRS and GCIPL PRS for any of the analysis performed (Supplementary Fig. 7).

#### Retinal and AD PRSs as predictors of dementia

We also assessed in the GR@ACE cohort whether dementia (all-type, ADD, and non-ADD) or dementia plus MCI status were predicted by the AD PRS, RNFL PRS (Fig. 3B), or GCIPL PRS (Fig. 3C) using adjusted logistic regression models (Supplementary Table 8, Supplementary Fig. 8). As expected, the risk of all-type dementia increased with increasing AD PRS scores (*β* = 0.04, *p* = 8.5e−50); this effect remained significant in the non-ADD group (*β* = 0.01, *p* = 5.0e−6), although it was smaller in this group than in the ADD cohort (*β* = 0.03, *p* = 5.4e−26), probably due to the fact that the study from Bellenguez et al. included not only ADD, but also other types of related dementia, offering a broader spectrum for genetic associations. By contrast, neither the RNFL PRS nor the GCIPL PRS were associated with the risk of dementia, with the exception of the GCIPL PRS and all-type dementia (*β* = 0.01, *p* = 3.0e−2) and for dementia plus MCI (*β* = 0.01, *p* = 4.5e−2). Moreover, none of the SNPs reported by Currant et al. were associated with dementia, apart from two SNPs in chromosome 6: rs9398171 within the FOXO3 and KIF6 genes, conferring a decreased and increased risk, respectively, for all-type dementia, although none passed the multiple testing correction (Supplementary Tables 9–10, Supplementary Figs. 9–10).

## Discussion

This article presents the results of a genome-wide association study for macular RNFL and GCIPL thickness on more than 3000 individuals with different degrees of cognitive decline (CH, MCI, and different types of dementia, mostly ADD). We identified several genetic *loci* affecting retinal thickness, some previously associated with these structures or related phenotypes that were replicated across eyes and OCT cohorts. Both retinal measures showed a strong correlation, but only GCIPL thickness showed evidence of association with all-type dementia. We assessed genetic overlap between cognitive status and retinal thickness using RNFL and GCIPL PRSs as predictors of dementia (all-type, ADD, and non-ADD), but only a weak association was observed between GCIPL PRS and all-type dementia. On the other hand, AD PRS was not a predictor of RNFL or GCIPL thickness, despite three ADD candidate SNPs’ being associated with RNFL (rs587709, within the LILRB2 gene) and GCIPL (rs7384878, within the SPDYE3 gene, and rs6586028, within the TSPAN14 gene).

Several studies have reported significant RNFL thinning in AD compared to CH individuals, while others have not (reviewed by Majeed et al. [44]), but, as retinal thickness decreases with age, it is difficult to establish a causal relationship between both events. In fact, unadjusted analyses from our dataset showed that AD cases had thinner RNFL and GCIPL than the CH and MCI subjects, but this difference was not observed after adjustment for confounding variables. Only a small reduction in the GCIPL thickness in dementia patients was observed, but this association was no longer significant when only patients with ADD were considered, in accordance with previous results from our group [12, 45].

Mendelian randomization (MR) uses genetic data as an instrumental variable for testing the association between the exposure of interest (GCIPL and RNFL thickness) and the study outcome (dementia). To our knowledge, this was the first time MR was applied to explore this association, and our results do not support the existence of common genetic factors for retinal thickness and dementia, either of AD or non-AD dementia types. A recent report by Sekimitsu et al. [46] in the UK Biobank population described a thicker inner nuclear layer (INL), chorio-scleral interface (CSI), and inner plexiform layer (IPL) among individuals with higher AD PRS scores, although the observed effect was very weak, below our statistical power; moreover, neither the individual association of the AD SNPs, nor the association of retinal PRSs and AD, were explored to fully clarify the relationship between retinal thickness and dementia.

Another potential limitation of our study would be the differences observed between retinal measures generated by the OCT Maestro and Triton devices. These outputs are determined by a complex combination of technical parameters differing by vendors and OCT technicians that may result in discordant estimations of retinal layer thickness [47]. To minimize this effect, both datasets were analyzed independently, and the association results were combined using meta-analysis. Moreover, only signals showing concordant effects in both eyes were considered.

Another interesting conclusion from our study is the suggestion, derived from the adjusted regression models for AD PRS as a predictor of dementia, that AD and non-AD dementia share genetic factors, as previously described by other authors [48].

## Conclusions

Our results do not support the existence of a genetic link between dementia and retinal thickness, as suggested by the lack of association of AD genetic risk factors with macular RNFL and GCIPL measures, and, conversely, of RNFL and GCIPL genetic determinants with dementia, potentially limiting the utility of OCT measures as early biomarkers of cognitive decline. Reported associations between retinal phenotypes and cognitive status may occur via non-genetic risk exposures or may be diluted because are mediated by a common risk factor (for example diabetes). These mechanisms are probably complex enough (due to multiple environmental and genetic factors) that they cannot be disentangled using our current sample size. Much larger efforts are necessary to dissect such complex relationships.

### Supplementary Information

**Additional file 1: Supplementary Figures 1-10**.

**Additional file 2: Supplementary Tables 1-10**.

## Data Availability

Available upon reasonable request.

## Abbreviations

AD: Alzheimer’s disease
ADD: Alzheimer’s disease dementia
CH: Cognitively healthy
Dp: Dioptres
ETDRS: Early Treatment of Diabetic Retinopathy Study
FACEHBI: Fundació ACE Healthy Brain Initiative
GCIPL: Ganglion cell inner plexiform layer
GCL: Ganglion cell layer
GO: Gene Ontology
GWAS: Genome-wide association study
GR@CE: Genome Research at Fundació ACE
IOP: Intraocular pressure
MAF: Minor allele frequency
MCI: Mild cognitive impairment
MR: Mendelian randomization
NORFACE: Neuro-Ophthalmology Research at Fundació ACE
OCT: Optical coherence tomography
PC: Principal component
QC: Quality control
PRS: Polygenic risk score
RNFL: Retinal Nerve fiber layer
SCD: Subjective cognitive decline
SNP: Single-nucleotide polymorphism
SS: Swept source
TABS: Topcon Advanced Boundary Segmentation TM
