# Supplementary material for: Genome-wide association study and polygenic risk scores of retinal thickness across the cognitive continuum: data from the NORFACE cohort
Source: Alzheimers Res Ther. 2024 Feb 16;16:38. doi: 10.1186/s13195-024-01398-8 (PMC10870444; doi:10.1186/s13195-024-01398-8)

Supplementary figures.

Supplementary figure 1. Correlogram of GCIPL and RNFL thickness measures of participants with data from both OCT Triton and Maestro datasets (N=582).

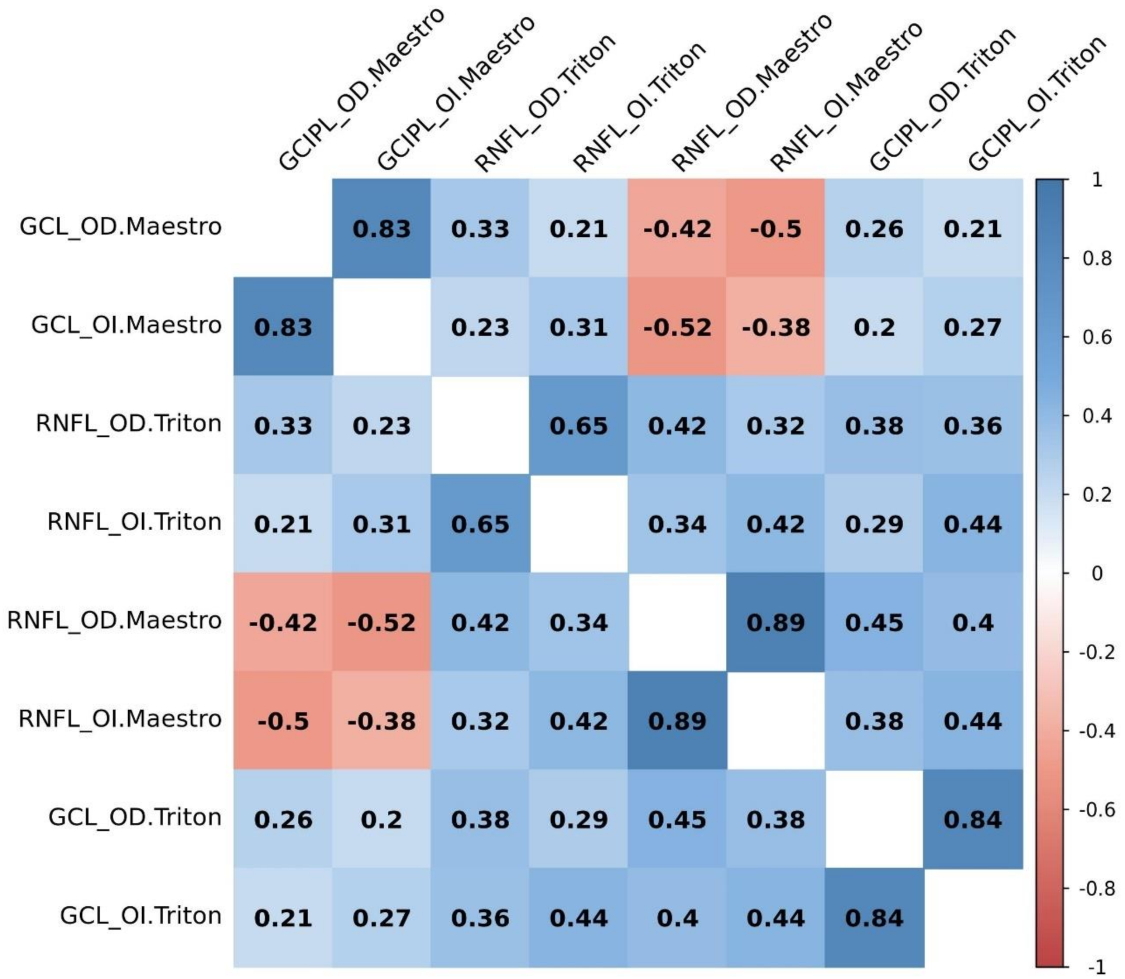

OD: right eye; OI: left eye.

Supplementary figure 2. Correlations between retinal PRSs and AD PRS vs RNFL/GCIPL thickness.

A

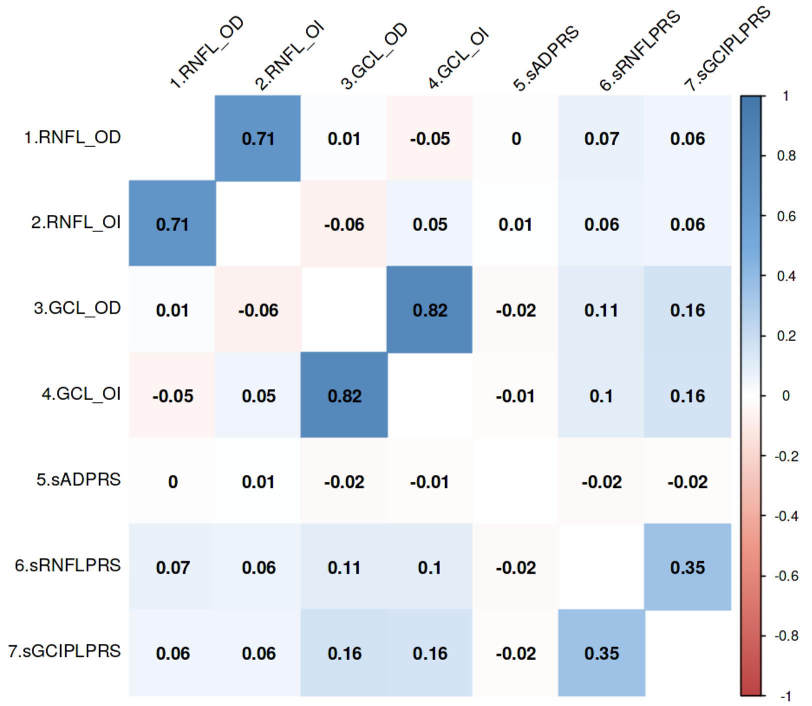

B

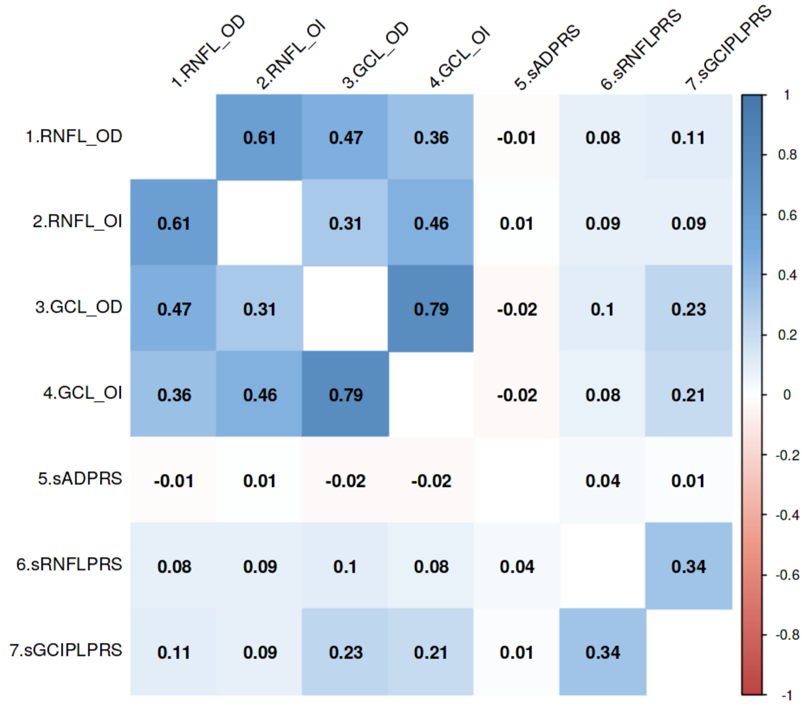

Supplementary figure 3. Adjusted logistic regression models for AD PRS, GGCIPL PRS and RNFL PRS as predictors of RNFL and GCIPL thickness (Maestro & Triton OCT cohorts).

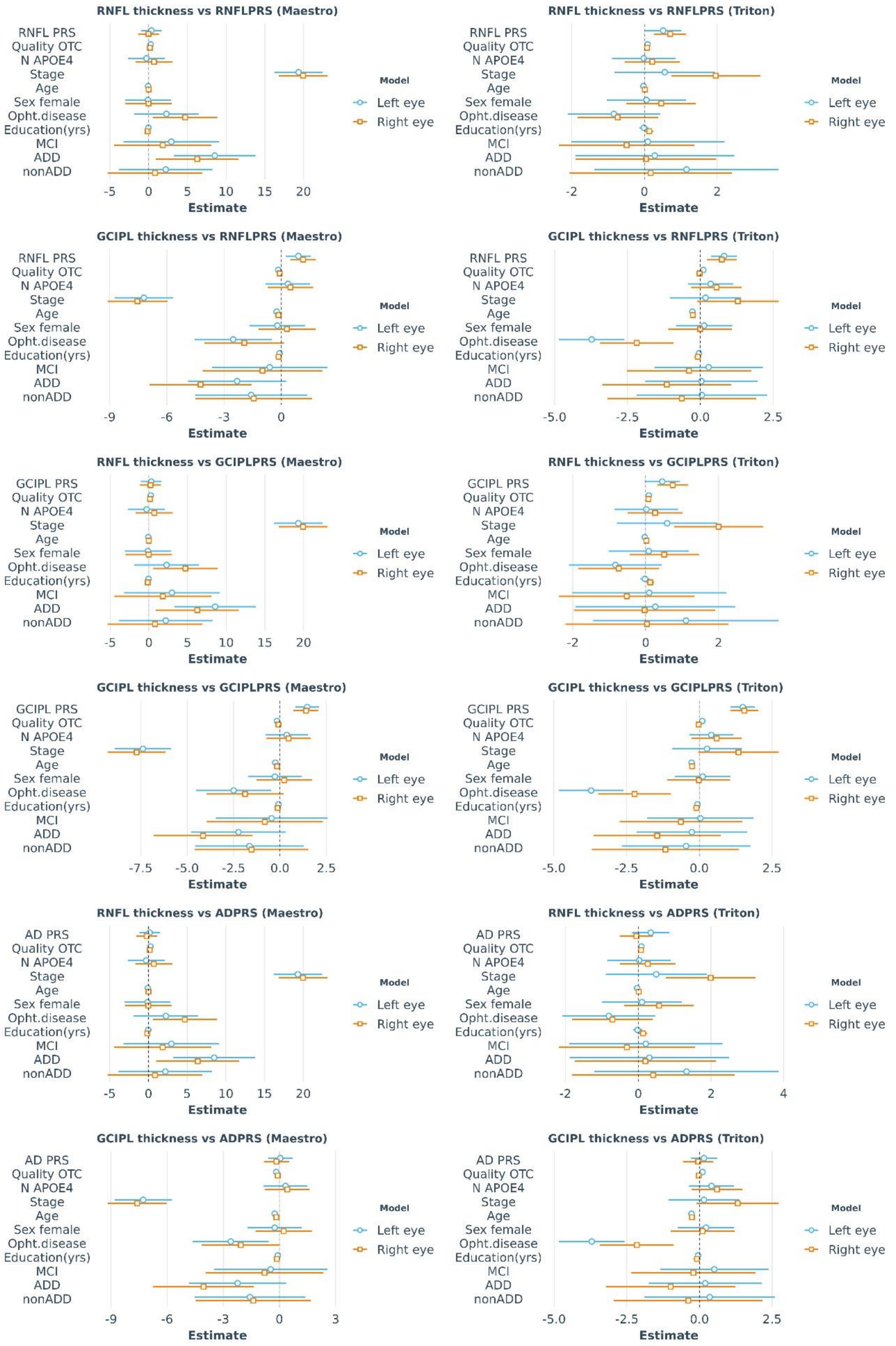

Supplementary figure 4. Adjusted logistic regression models for AD PRS SNPs as predictors of RNFL and GCIPL thickness (Maestro OCT cohort).

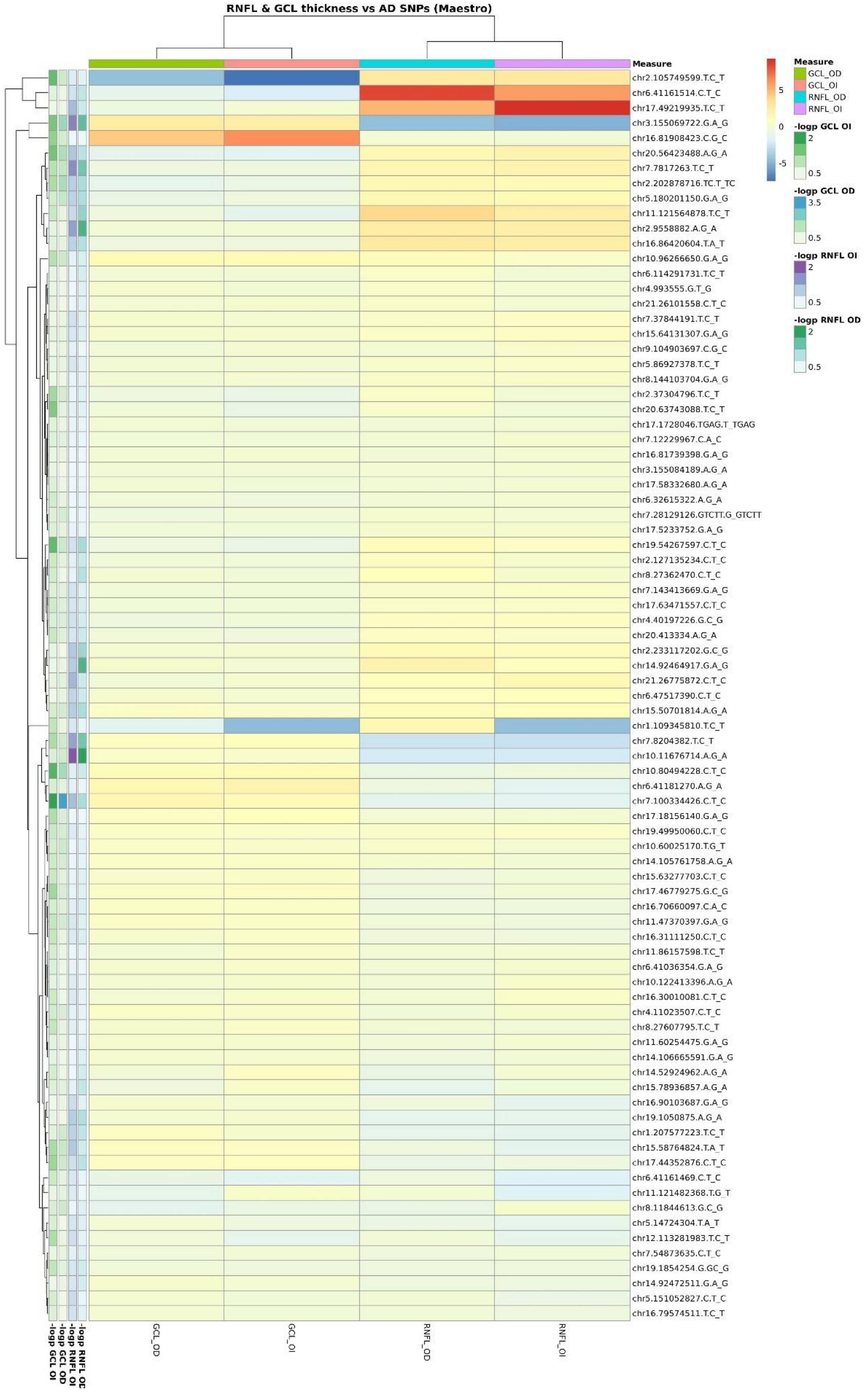

OD: right eye; OI: left eye.

Supplementary figure 5. Adjusted logistic regression models for AD PRS SNPs as predictors of RNFL and GCIPL thickness (Triton OCT cohort).

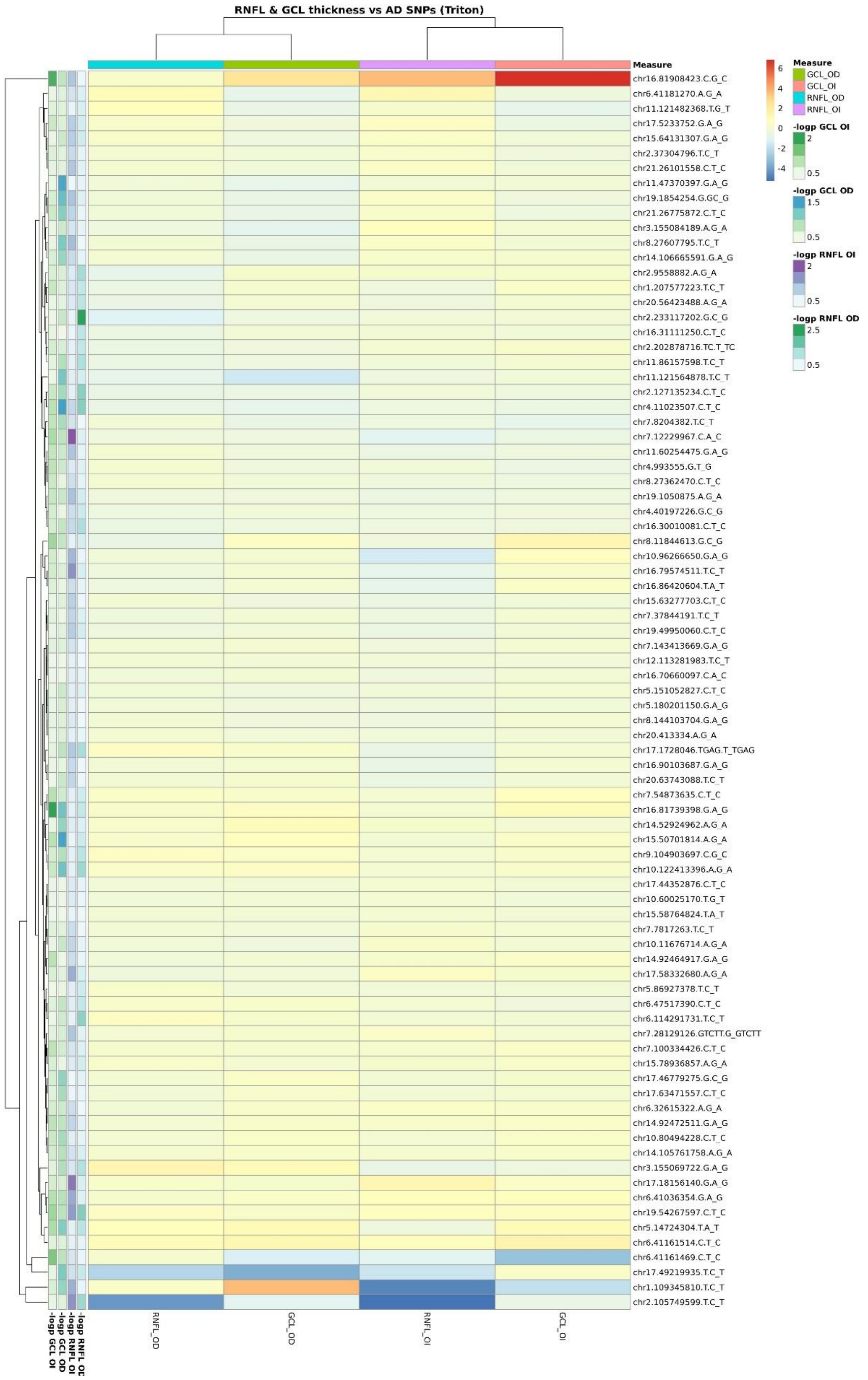

OD: right eye; OI: left eye.

Supplementary figure 6. Adjusted logistic regression models for AD PRS SNPs as predictors of RNFL and GCIPL thickness (meta-analysis).

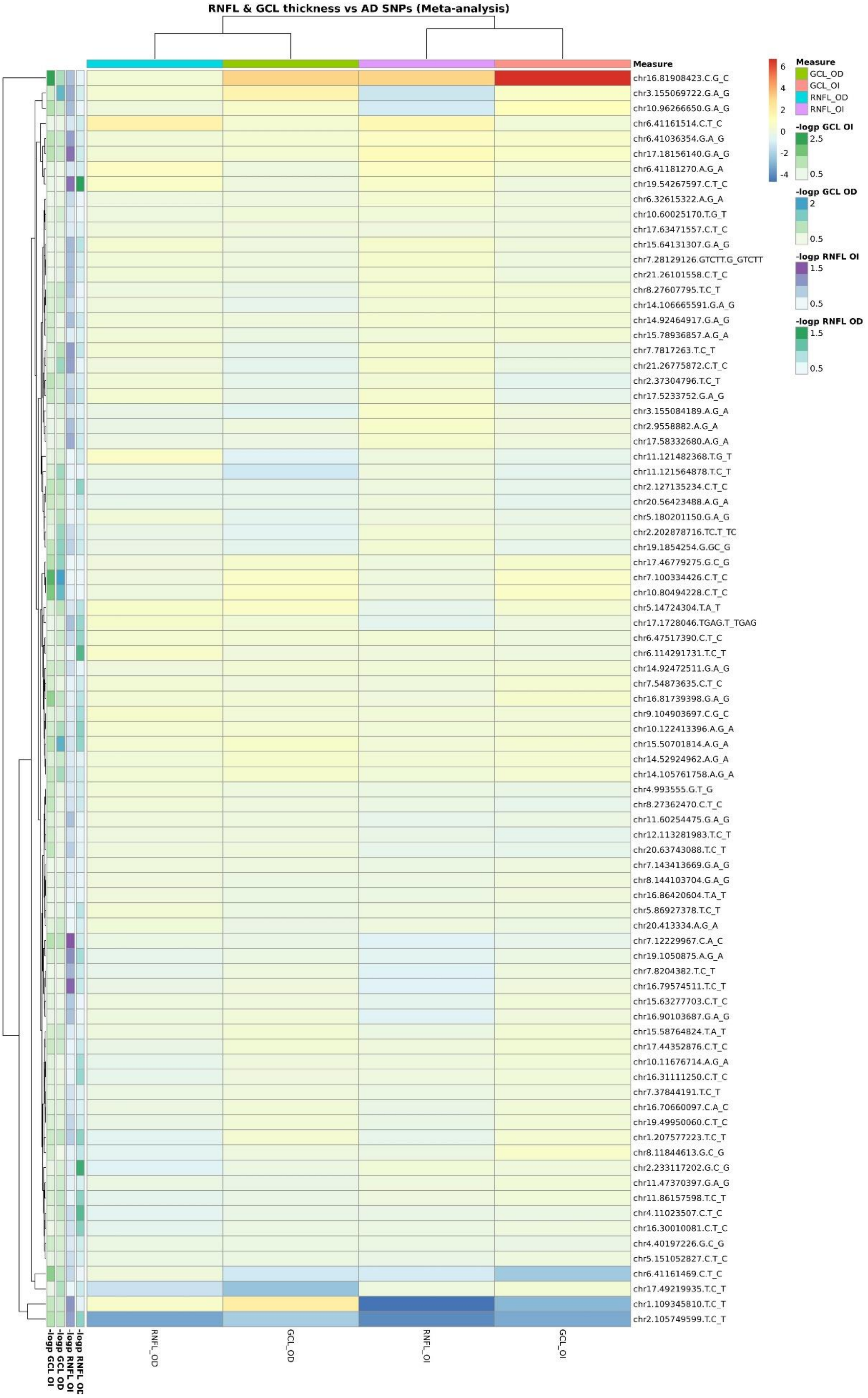

OD: right eye; OI: left eye.

Supplementary figure 7. Correlations between retinal PRSs and AD PRS in the GR@ACE cohort.

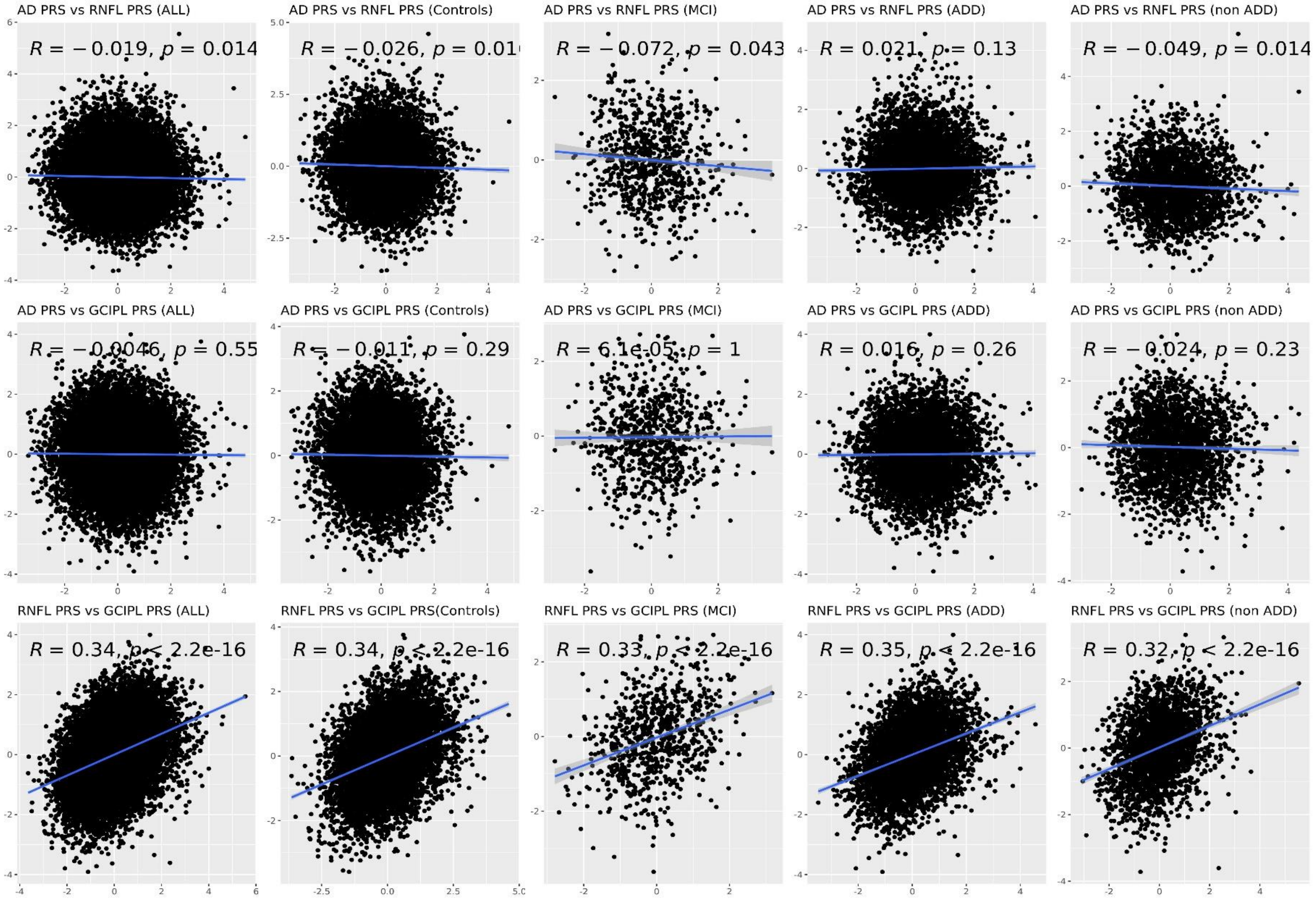

Supplementary figure 8. Adjusted logistic models for RNFL PRS, GCIPL PRS and AD PRS as predictors of dementia (all-type, ADD, non-ADD).

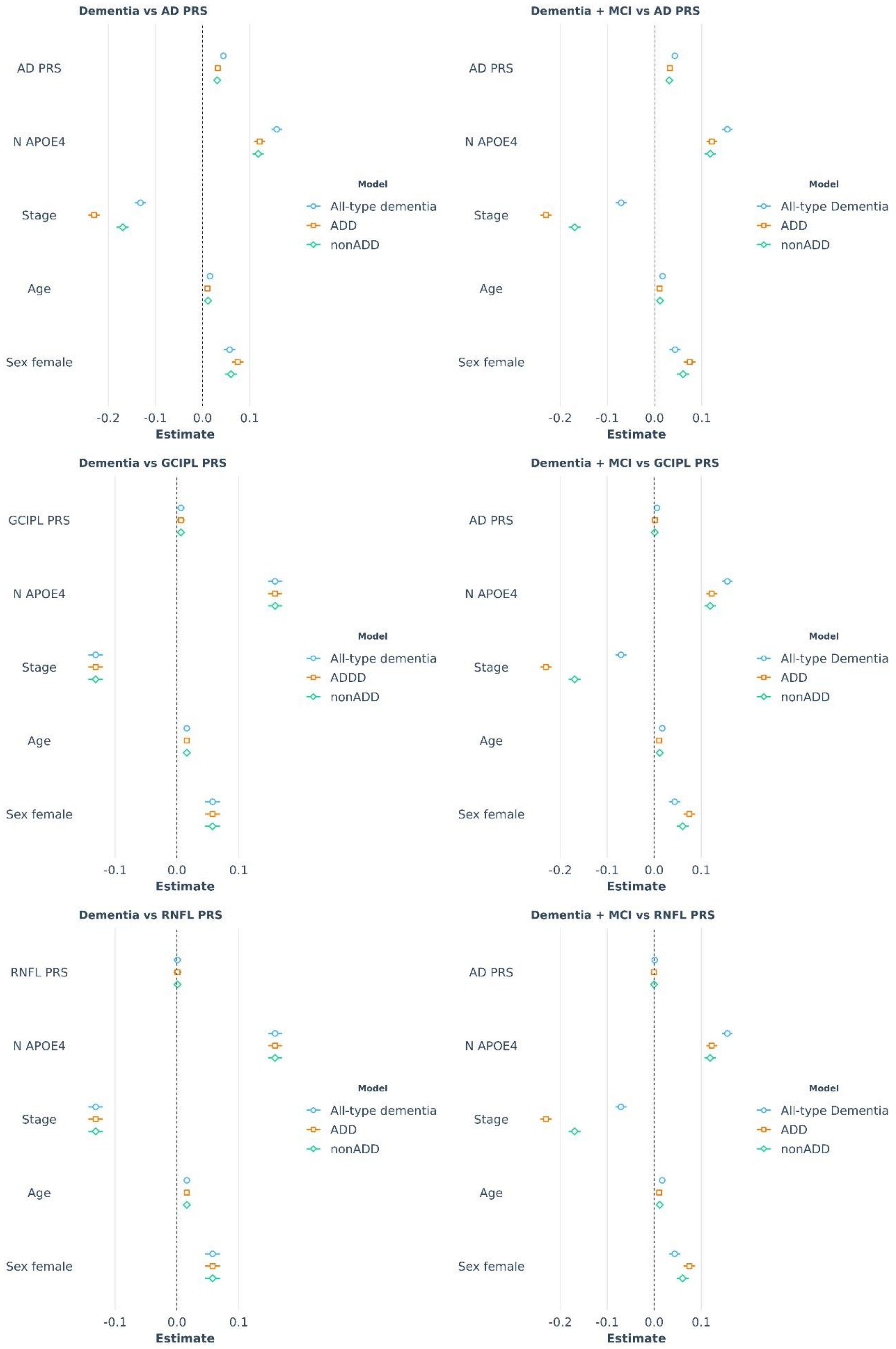

Supplementary figure 9. Adjusted logistic regression models for RNFL PRS SNPs as predictor of dementia.

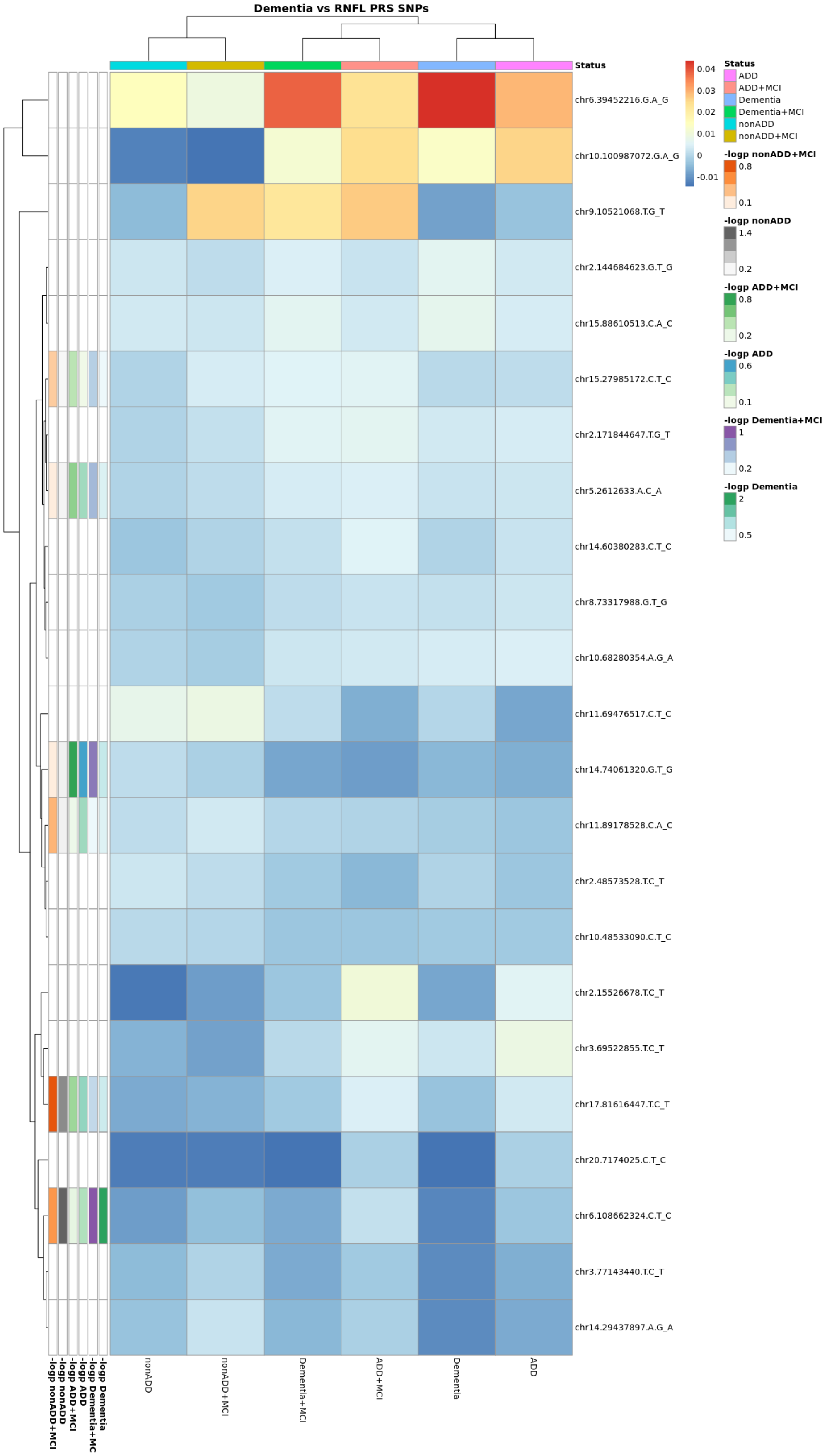

Supplementary figure 10. Adjusted logistic regression models for GCIPL PRS SNPs as predictor of dementia.

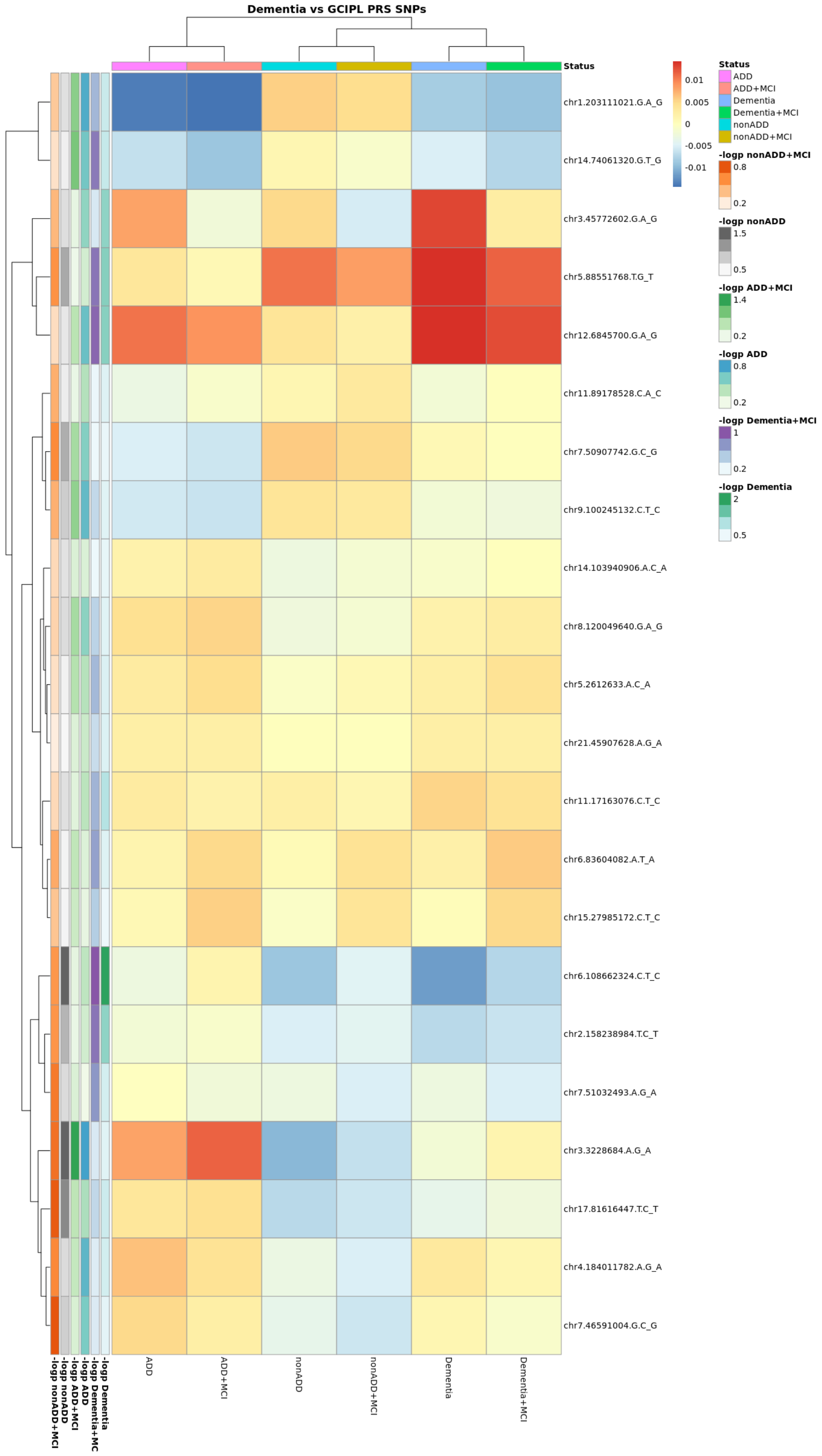

Supplement: Supplementary file 1 — Additional file 1: Supplementary Figures 1-10. [file 13195_2024_1398_MOESM1_ESM.pdf]
